# Supplementary material for: GPR64, Screened from Ewing Sarcoma Cells, Is a Potential Target for Antibody-Based Therapy for Various Sarcomas
Source: Cancers (Basel). 2022 Feb 5;14(3):814. doi: 10.3390/cancers14030814 (PMC8834492; doi:10.3390/cancers14030814)

Cancers

Koichi Nakamura

Figure S1：Uncropped western blot figure of Figure 4

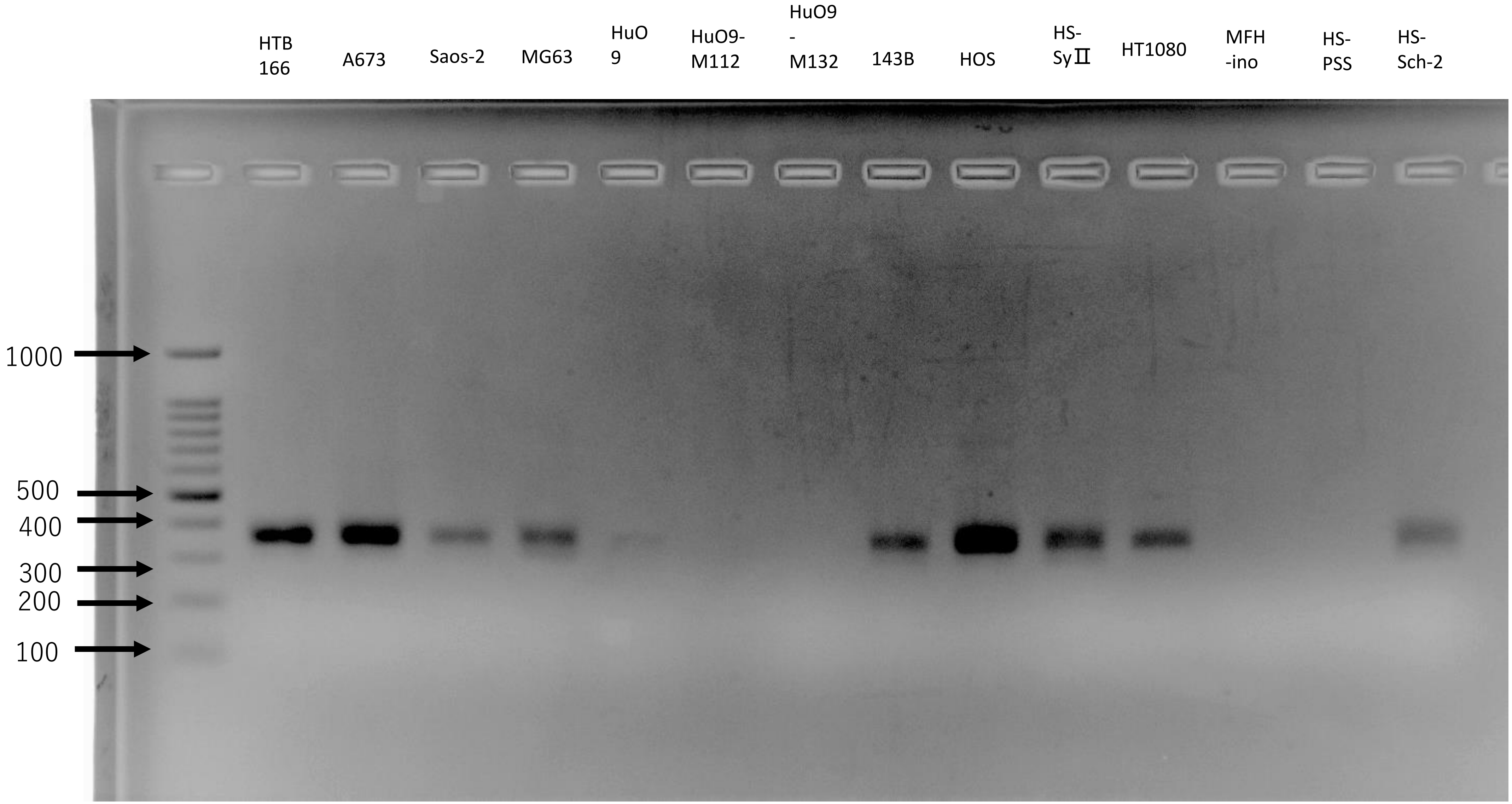

Figure S1：Uncropped western blot figure of Figure 4

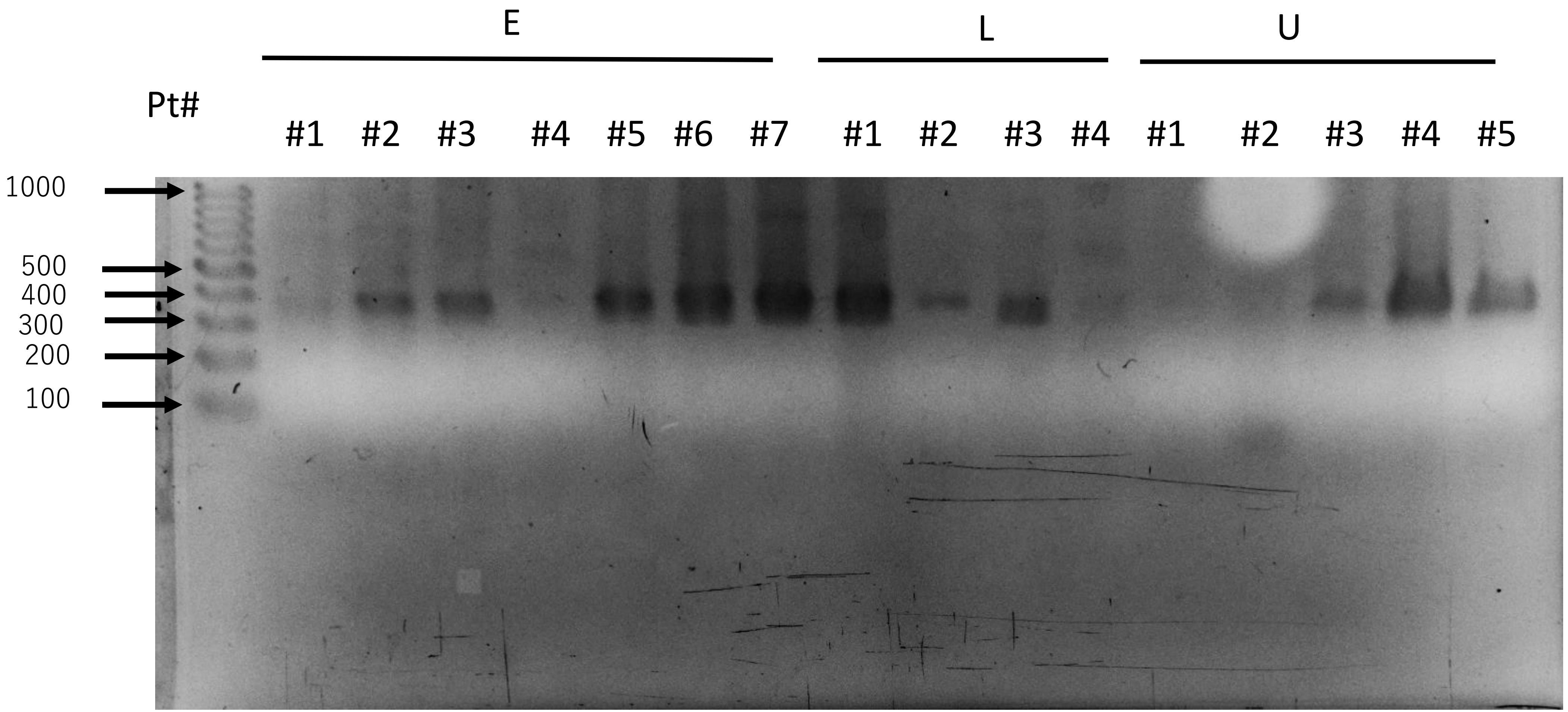

Figure S1：Uncropped western blot figure of Figure 4

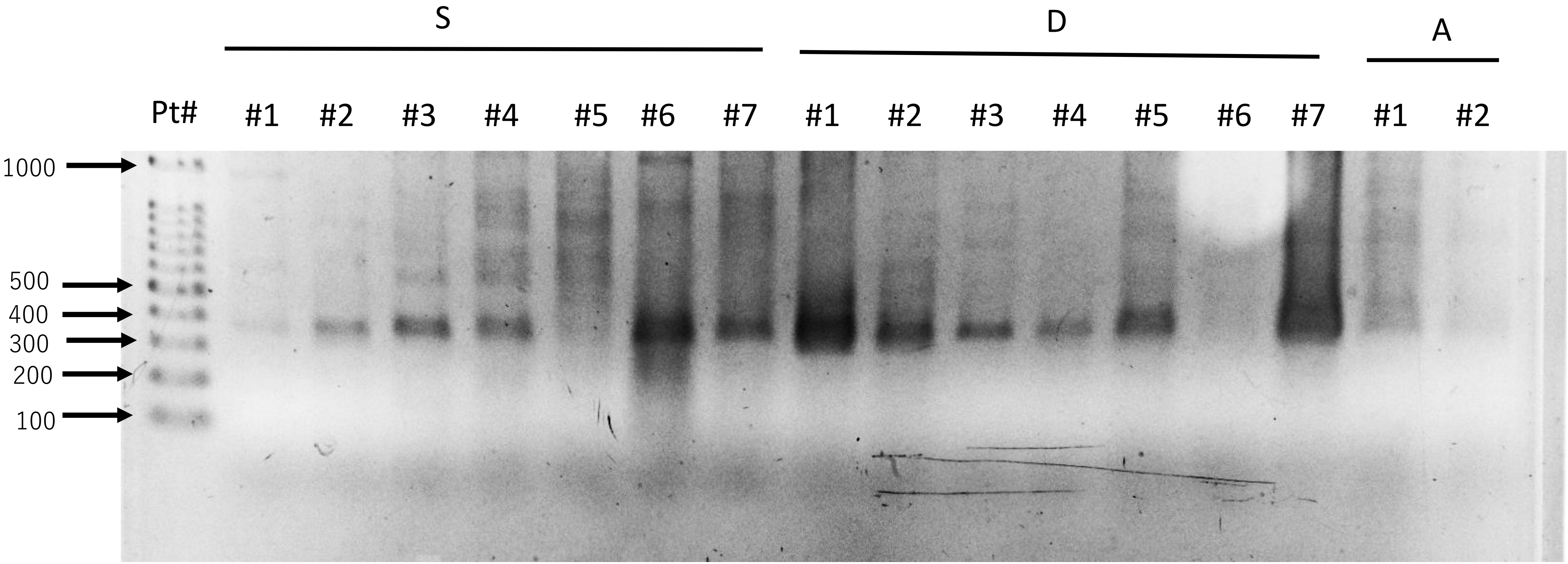

Figure S2：Uncropped western blot figure of Figure 5

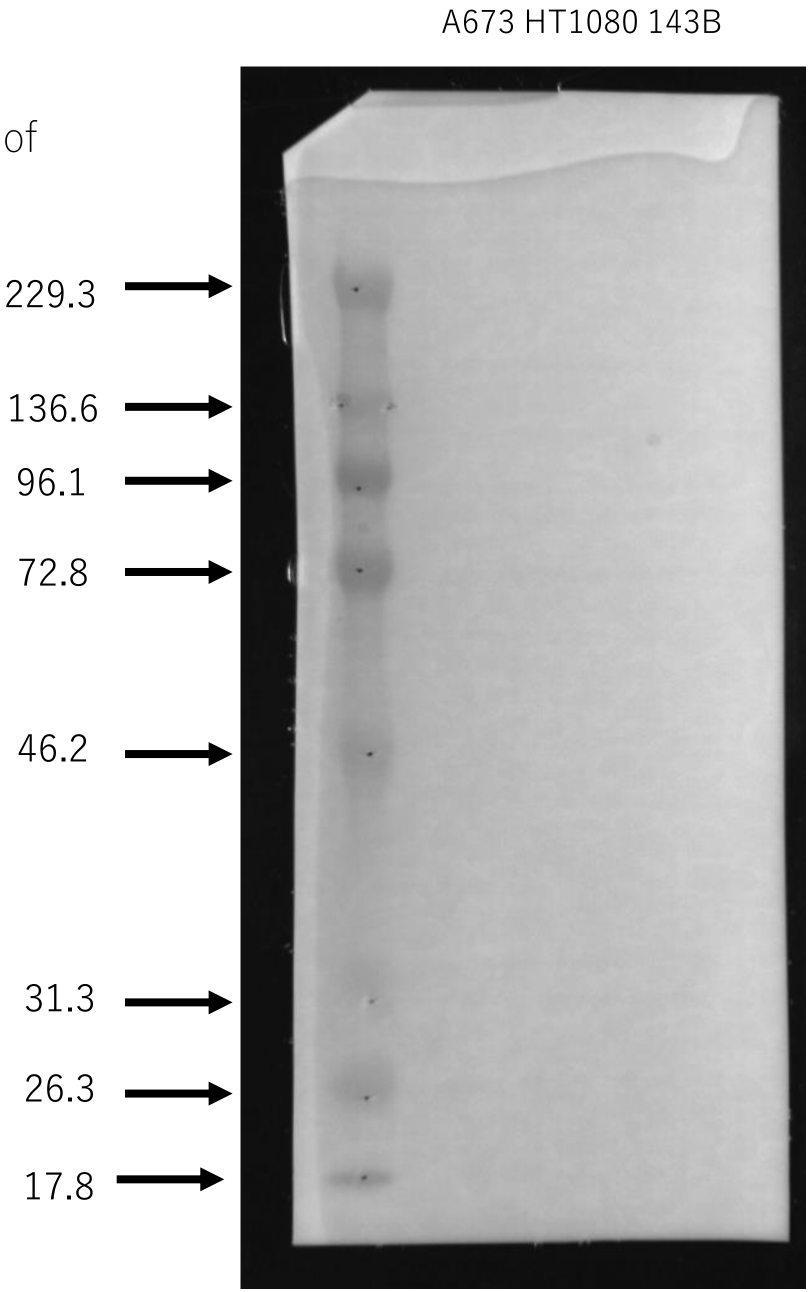

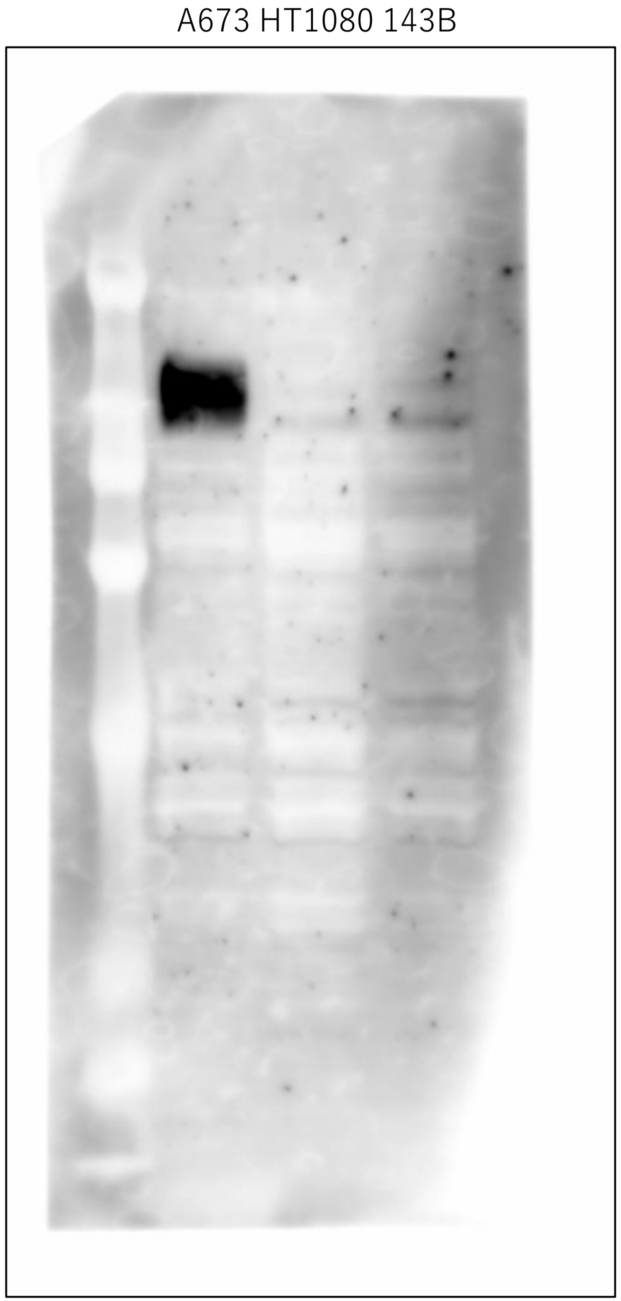

Figure S2：Uncropped western blot figure of Figure 5

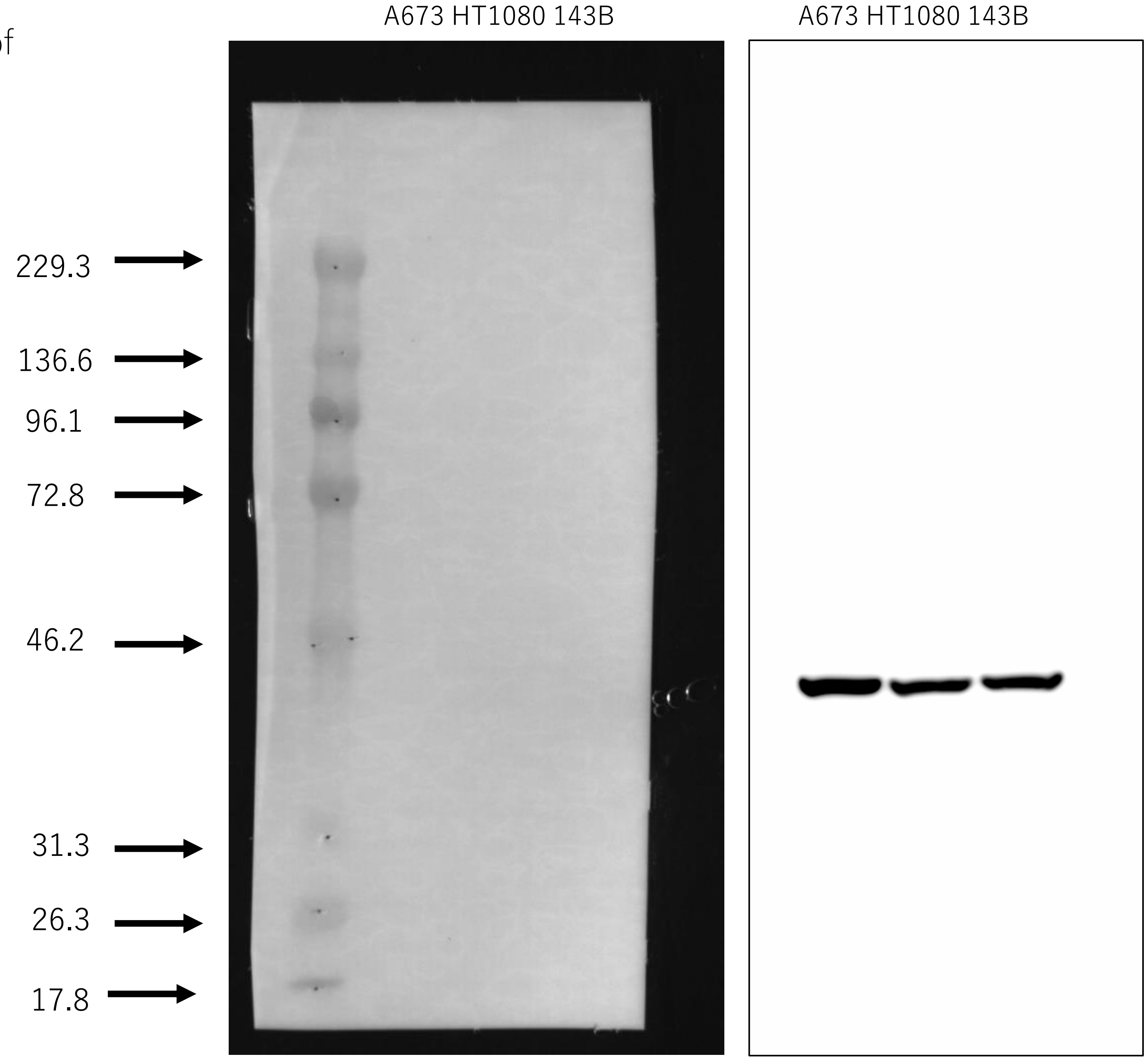

Supplement: Supplementary file 1 [file cancers-14-00814-s001.zip › cancers-1573126-supplementary.pdf]
